# Intragenogroup Recombination in the Complete Genome Sequence of Human Sapovirus Circulating in Bangladesh

**DOI:** 10.1128/genomeA.00388-18

**Published:** 2018-05-03

**Authors:** Shuvra Kanti Dey, Mariya Kibtiya Sumiya, Modhusudon Shaha, Razoanul Haque, Shoko Okitsu, Hiroshi Ushijima

**Affiliations:** a Department of Microbiology, Jahangirnagar University, Savar, Dhaka, Bangladesh; b Division of Microbiology, Department of Pathology and Microbiology, Nihon University School of Medicine, Tokyo, Japan; c Microbial Biotechnology Division, National Institute of Biotechnology, Savar, Dhaka, Bangladesh

## Abstract

Human sapovirus (SaV) is responsible for severe gastroenteritis among infants and children. Research about the genetic configuration of SaV is scarce in Bangladesh. Here, we report the complete genome sequence of an SaV strain with intragenogroup recombination, isolated from an infant with severe diarrhea in Bangladesh in 2005.

## GENOME ANNOUNCEMENT

Human sapovirus (SaV) is a single-stranded, positive-sense RNA virus that causes gastrointestinal tract infections, which is one of the major causes of gastroenteritis, responsible for substantial morbidity and mortality worldwide (1). Globally, about 2.2 million people, including children, die annually due to the infections caused by gastrointestinal viruses (2), of which SaV plays a significant role, after rotavirus and norovirus (3). Furthermore, Bangladesh also bears an SaV disease rate of about 2.7% among infants and children with acute gastroenteritis (4).

SaV is a member of the family Caliciviridae and is composed of a genome of approximately 7,100 to 7,700 bp in size (5). The genome primarily encodes two functional open reading frames (ORFs), ORF1 and ORF2. Another ORF, ORF3, is also detected in human hosts, with unidentified function (6). According to the literature, a viral polyprotein (nonstructural protein, NS) and the major capsid protein (VP1) are encoded by ORF1. Furthermore, ORF2 and ORF3 encode the minor capsid protein (VP2) and small basic protein, respectively (6). Based on the genome sequence, human SaV has multiple genetic clusters, including four genogroups (GI, GII, GIV, and GV) with 17 genotypes (GI.1 to GI.7, GII.1 to GII.7, GIV.1, GV.1, and GV.2) (6, 7). Among them, GI and GII are the most predominant genogroups worldwide (8). Different evidence showed that the molecular evolution of SaV occurred in intergenogroup and intragenogroup recombination (9). Here, we report the complete genome sequence of a human SaV strain, isolated from an infant with severe diarrhea in Bangladesh in 2005.

The stool sample collected from the patient was diluted with 10% phosphate-buffered saline (PBS) and centrifuged at 5,000 rpm for 10 min. Viral RNA was extracted using a QIAamp viral RNA minikit (Qiagen, Germany). SaV cDNA was synthesized from RNA using a cDNA kit (Applied Biosystems, USA), and the complete genome was amplified by PCR, using eight sets of primer pairs spanning the entire genome. The amplicons were then purified using a Qiagen PCR purification kit (Qiagen, Germany) and sequenced using a BigDye Terminator version 3.1 cycle sequencing kit (Applied Biosystems, USA). The overlapping fragments were assembled using SeqMan II (DNAStar, Madison, WI). The phylogenetic and recombination analyses were performed using MEGA6 (10) and RDP4 (http://web.cbio.uct.ac.za/~darren/rdp.html), respectively.

The assembled genome of SaV comprises 7,458 nucleotides and belongs to the genogroup GI.1/2 recombination. The genome was found to be highly recombinant, with intragenogroup recombination, in which the nonstructural protein-encoding and VP1-encoding regions showed genogroups GI.1 and GI.2, respectively. This study might help clinicians to understand the evolutionary pattern of SaV in human population and find a proper treatment strategy.

### Accession number(s).

The complete nucleotide sequence of SaV isolate Hu/BD/697/2005/BGD was deposited in GenBank under the accession number GQ261222.
